# Stable protease from *Bacillus licheniformis*-MA1 strain: statistical production optimization, kinetic and thermodynamic characterization, and application in silver recovery from used X-ray films

**DOI:** 10.1186/s12934-025-02706-z

**Published:** 2025-05-05

**Authors:** Mohamed A. A. Abdella, Samia A. Ahmed

**Affiliations:** Chemistry of Natural and Microbial Products Department, Pharmaceutical and Drug Industries Research Institute, National Research Centre, Dokki, Cairo, 12622 Egypt

**Keywords:** Bio-additive detergent, Multi-factorial designs, Enzyme stabilities, Thermodynamics

## Abstract

**Background:**

Alkaline proteases are useful enzymes for various industrial applications as bio-additives in detergents and in the recovery of silver from used X-ray films. Therefore, many strategies were used to increase enzyme production and reduce production costs by using microbial cultures, using agro-industrial waste, and improving growth conditions via statistical methods. The enzyme kinetics and thermodynamics were studied as well as its ability to recover silver was also evaluated.

**Results:**

An alkaline protease suitable for industrial applications was produced by *Bacillus licheniformis* strain-MA1. The ability of *B. licheniformis* strain-MA1 to produce protease was optimized using multi-factorial designs (Plackett–Burman and Box–Behnken). Optimization process improved enzyme production by 9.6-fold over that obtained from the original medium. Highest alkaline protease production was reached after 72 h at pH 7.0, 35 °C, and 150 rpm. The protease was maximally active at 50 °C and pH 9.0 with high thermal and pH stability. The protease showed high catalytic efficiency and high affinity toward substrate with low activation energy (*Ea*). In addition, the thermodynamic parameters of protease enzyme (enthalpy, free energy, and entropy) were also investigated and showed its superior thermal stability. At 70 °C the thermal deactivation constant (*kd*) was 4.75-fold higher than that at 50 °C. The higher *t*_*0.5*_, *D*-values, and activation energy for thermal denaturation (*Ed*) of the protease indicated its higher thermal stability and thus its potential application in industrial processes. The compatibility of the protease with laundry detergents at 40 °C was higher than at 50 °C. In the presence of EDTA, the protease enzyme retained 93.6% of its activity. Furthermore, the crude enzyme successfully hydrolyzed the gelatin layer from X-ray films waste after 1 h enabling recycling and reuse.

**Conclusions:**

Stable alkaline protease from *B. licheniformis* strain-MA1 was suitable for some industrial aspects as a bio-additive in detergents and capable of recovering silver from used X-ray.

## Introduction

Alkaline proteases are a group of enzymes that show optimal activity and stability at alkaline pH and are widely utilized in different industries [1]. They are very useful as cleaning agent, meal preparation, hair removal agents, health centers and environmental protection [2]. Sales of alkaline proteases represent 35% of total commercial proteases [3].

Silver has significant industrial and economic applications and is often used in the manufacture of jewelry, silverware, electronic equipment, and dental fillings [4]. Nearly two billion X-ray images are taken each year and these X-ray sheets are good sources of silver in their gelatinous layer [5]. Silver recovery by burning films leads to environmental pollution and health risks [6]. Since the silver is bound to gelatin, it has been demonstrated that proteases with gelatinase activity can be used to recover silver from X-ray films waste. Gelatin hydrolysis by enzyme helps in extract the silver and the polyester base layer can be recycled [7].

In addition, proteases are standard bio-additives of all detergents kinds as they can remove all kinds of protein stains such as blood, and milk [3, 8]. The bio-detergent is mostly composed of enzyme produced by bacteria and it is known as bacterial enzyme detergent [2]. The following properties allow protease to be used as a bio-additive in detergent: (a) stability at high temperatures, (b) activity and stability at alkaline pH, and (c) compatibility with detergent components [2]. Thermostable alkaline protease has been widely used for application in harsh conditions [1].

Proteases are produced using plant, animal and microbial sources. While microbial sources are preferred commercially due to their rapid production, environmental friendliness, economical production, and potential for improved production [9–11]. Microorganisms are also the preferred source of alkaline protein due to their maintenance and genetic transformation. They are not affected by seasonal changes, and can be produced on a large scale using industrial agricultural waste to decrease the costs of production and environmental pollution [3, 12]. Moreover, microbial enzyme production of desired industrial products is regular and abundant, and its shelf life is long, which means that once this enzyme is isolated and purified, it can be stored for a long time with high stability and efficacy [2].

One of the major commodity agricultural products across the globe is oilseeds. The seed components remaining after oil extraction (industrial residues) are called oil cakes such as sesame cake. Such oil cakes are composed of proteins and carbohydrates and commonly used as fertilizer or animal feed [10]. In addition, these residues can be used as a substrate for microbial growth and enzyme production.

Several strategies have been used to increase enzyme production and reduce production costs including the use of microbial cultures, use of agro-industrial waste, and optimize the growth conditions via statistical methods [13].

An experimental design incorporating response surface methodology was used to address nutritional issues for protease-producing microorganisms, where priority is given to meeting important requirements of the microorganisms [9].

The objectives of this study were to improve protease production by the *B. licheniformis* strain-MA1 using Plackett–Burman and Box–Behnken statistical designs, and to characterize the stability, kinetic, and thermodynamic parameters of the produced protease. Finally, evaluate the ability of the protease to hydrolyze and recover gelatin from X-ray films waste.

## Materials and methods

### Materials

Sesame cake was obtained from Fats and Oils department, National Research Centre, Egypt. Trichloroacetic acid (TCA) and casein were obtained from Sigma Chemical Co. [St. Louis, MO, USA]. Tryptone, dextrose, agar, and yeast extract were supplied from Merck (Darmstadt, Germany). Folin reagent was purchased from SDFCL Sd fine-Chem limited, Mumbai, IndiaMART. Skim milk powder was from Sigma Chemical Co. [St. Louis, MO, USA]. All chemical reagents of high quality were used.

### Methods

#### Bacterial strain and screening for proteolytic activity

The bacterial strain isolated and identified as *B. licheniformis* strain-MA1 [14] was used for protease production. Using the streaking manner, the bacterial strain was plated on sterilized skimmed-milk agar (SMA) plate medium [15] consisting of the following components (g%): dextrose 0.1, skim milk powder 2.8, tryptone 0.5, yeast extract 0.25, and agar 1.5 at pH 7.0. After incubation for 24 h at 35 °C (in an inverted position), proteolytic activity was recognized by the clear area appearance around the bacterial colonies.

#### Estimation of enzyme activity

The protease activity was estimated using casein as substrate [3, 4]. The reaction was prepared by adding enzyme (0.5 ml) to 0.5 ml casein (1% in 0.1 M sodium phosphate buffer pH 7.0). The reaction was incubated in a water bath at 40 °C for 20 min. To stop the reaction, 1 ml of 10% TCA was added and left for 10 min for precipitation at room temperature. Then, the mixture was centrifuged at 10,000×*g* for 15 min to get the clear supernatant containing hydrolyzed proteins that were assessed according to Lowry et al. [16] method. All measurements were performed in triplicates and the results were the mean ± standard deviation. One unit of the enzyme activity (U) was defined as the enzyme quantity which releases 1 μg tyrosine/min under the assay conditions.

#### Examination of diverse media for protease production

The bacterial inoculum was prepared by transferring a loop-full from *B. licheniformis* strain-MA1 slant into a 20 ml of sterilized flask of Luria–Bertani (LB) broth, and then incubated at 35 °C under 150 rpm. After 24 h, 1 ml of the bacterial inoculum was cultivated in 250-Erlenmeyer flasks (50 ml, pH 7) consisting of the following media individually (g/l):

- M1: glucose 10, peptone 5, yeast extract 5, KH_2_PO_4_ 1, MgSO_4_·7H_2_O 0.2 [17].
- M2: glucose 10, peptone 5, KH_2_PO_4_ 0.02, MgSO_4_·7H_2_O 0.2, CaCl_2_ 0.4 [18].
- M3: peptone 5, yeast extract 5, KH_2_PO_4_ 5, FeSO_4_·7H_2_O 0.1, CaCl_2_ 0.2, NaCl 3 [19].
- M4: glucose 10, casein 5, yeast extract 5, KH_2_PO_4_ 2, MgSO_4_·7H_2_O 2 [20].
- M5: corn husk 10, lactose 2.5, casein 15, KH_2_PO_4_ 1, MgSO_4_·7H_2_O 0.5, CaCl_2_ 2 [12].

The cultivated flasks were incubated at 35 °C and 150 rpm for 48 h, then centrifuged at 4 °C and 10,000×*g* for 15 min and the supernatant was used as a crude protease.

#### Improving protease production using multi-factorial designs

##### Plackett–Burman design

Plackett–Burman (PB) design is considered a screening tool to show the importance of variables affecting enzyme production [21]. According to PB design, 11 independent variables (wheat bran, corn cob, sesame cake, peptone, yeast extract, KH_2_PO_4_, (NH_4_)_2_SO_4_, FeSO_4_·7H_2_O, CaCl_2_, culture pH, and incubation period) were examined at lower (−) and higher (+) levels. The number of experimental trials was determined on the basis of (T = n + 1) where, T is the trials number and n is the number of screened variables. Also, a first-order polynomial model was employed to explain the PB experimental design based on the following equation:

$${\text{Y}} =\upbeta _{0} + \Sigma\upbeta _{{\text{i}}} {\text{X}}_{{\text{i}}}$$

where Y (the response), β_0_ (the model intercept), β_i_ [the variable estimate (linear coefficient)], and X_i_ (the level of independent variable).

##### Box–Behnken design

Box–Behnken (BB) design estimates the most significant variables selected by PB design to obtain the highest protease production [22] using 3 optimization levels (−, 0, and +). Based on BB design, 15 experimental trials were constructed and the data was fitted to the following second-order polynomial model:

$${\text{Y}} =\upbeta _{0} + \Sigma\upbeta _{{\text{i}}} {\text{X}}_{{\text{i}}} + \Sigma\upbeta _{{{\text{ii}}}} {\text{X}}_{{\text{i}}}^{{2}} + \Sigma\upbeta _{{{\text{ij}}}} {\text{X}}_{{\text{i}}} {\text{X}}_{{\text{j}}}$$

where Y (the predicted response), β_0_ (the intercept term), β_i_ (the linear coefficient), β_ii_ (the squared coefficient), β_ij_ (the interaction coefficient), and X_i_, X_j_ (the independent variables).

#### Biochemical characterization of protease

##### Effect of different temperatures and pHs

The effect of different temperatures ranging from 20 to 70 °C on the protease activity was studied at 0.1 M and pH 7 using sodium phosphate buffer. The relative activity (RVA%) was calculated as follows:

1 $${\text{RVA}}\left( \% \right) = \left( {{\text{Observed}}\;{\text{activity}}/{\text{Initial}}\;{\text{activity}}} \right) \times {1}00$$

The activation energy of protease (*Ea* KJ/mol) was estimated from the slope of the Arrhenius plot (log of residual activity % versus absolute temperature in Kelvin × 10^3^) as follows:

2 $$Slope = - Ea/{2}.{3}\,{\text{R}}\quad \left( {{\text{R}}\;{\text{is}}\;{\text{the}}\;{\text{gas}}\;{\text{constant}}\;{8}.{314}\,{\text{J}}/{\text{mol}}/{\text{K}}} \right)$$

Optimum pH of protease was investigated over a pH range of 4–11 using 0.1 M of the following buffer systems [23]: citrate buffer (pH 4–6), sodium phosphate (pH 6–8) and glycine NaOH (pH 8–11).

##### Effect of diverse inhibitors and metal ions

The crude protease was incubated with 5 mM of the tested reagents [KCl, NaCl, CaCl_2_, MgSO_4_, ZnSO_4_·7H_2_O, CoCl_2_, MnCl_2_, CuSO_4_, FeSO_4_, EDTA (Ethylene Diamine Tetra Acetic), Urea, and SDS] for 20 min at 50 °C and the RVA (%) was determined under optimized assay conditions. Protease activity was expressed as 100% in the absence of any reagents.

#### Kinetic parameters of protease enzyme

The enzyme activity was examined using different casein concentrations from 4.5 to 36.2 mg/ml. The kinetic parameters Michaelis–Menten constant (*K*_*m*_) and maximum reaction velocity (*V*_*max*_) for protease were measured at optimum assay conditions [24].

#### Enzyme stabilities

##### pH stability

The pH stability of protease was checked using 0.1 M of the following buffer: citrate buffer (pH 5–6), sodium phosphate (pH 7–8) and glycine NaOH (pH 9–11) before enzyme assay for 30 and 60 min at 37 °C. The residual activities (RSA %) as follows:

3 $${\text{RSA}}\left( \% \right) = \left( {{\text{Final}}\;{\text{activity}}/{\text{Initial}}\;{\text{activity}}} \right) \times {1}00$$

##### Thermal stability

Protease thermal stability was determined by measuring the RSA (%) of the enzyme at different temperatures 50, 60, and 70 °C for 60 min before enzyme assay, with samples taken at 15 min intervals.

The Arrhenius diagram was used to calculate deactivation rate constant (*kd*/min) (Log RSA % as a function of time) at the temperature used for inactivation as follows:

4 $$Slope = - kd$$

The half-life time (*t*_*0.5*_ min) and the decimal reduction time (*D*-value min) were calculated as follows:

5 $$t_{0.5} = 0.{693}/kd$$

6 $$D{\text{ - value}} = {2}.{3}0{2}/kd$$

The protease activation energy for denaturation (*Ed* KJ/mol) was determined from Arrhenius plot of ln *kd* versus 1/Temperature in Kelvin (°*K*) as follows:

7 $$Slope = - Ed/{\text{R}}$$

The change in enthalpy, free energy, and entropy for thermal enzyme inactivation were calculated as mentioned by Mostafa et al. [25] as follows:

8 $${\text{Enthalpy}}\left( {\varDelta {\text{H}}^*} \right) = Ed{-}{\text{RT}}$$

9 $${\text{Free}}\;{\text{energy}}\left( {\varDelta {\text{G}}^*} \right) = - {\text{RT}}\;{\text{ln}}\left( {Kd.{\text{h}}/Kb.{\text{T}}} \right)$$

10 $${\text{Entropy}}\left( {\varDelta {\text{S}}^*} \right) = \left( {\varDelta {\text{H}}^* - \varDelta {\text{G}}^*} \right)/{\text{T}}$$

where T (the absolute temperature, °K), h (the Planck’s constant, 11.04 × 10^–36^ J min), and *Kb* (the Boltzman constant, 1.38 × 10^–23^ J/K).

#### Stability and compatibility with laundry detergents

The stability and compatibility of protease with various powdered commercial detergents (Ariel, Bonux, Extra, Lange, Oxi, Persil, and Tide) was studied [3, 7]. Detergent solutions were prepared in tap water at concentrations of 1% (w/v). The present proteases in the laundry detergents were inhibited by heating at 95 °C for 10 min before enzyme addition. The protease was added to the detergent solution in a ratio of 1:1 (v/v), and then pre-incubated at 40 and 50 °C for 60 min prior to addition of the substrate. Preparation without commercial detergent was used as control and enzyme activity scored as 100%. The residual activities were calculated with reference to control (100% activity).

$${\text{The}}\;{\text{residual}}\;{\text{activities}}\left( \% \right) = \left( {{\text{activity}}\;{\text{with}}\;{\text{detergents}}/{\text{activity}}\;{\text{without}}\;{\text{detergents}}} \right) \times {1}00$$

#### Application of protease in the gelatin hydrolysis and recovery of silver from X-ray film waste

The ability of alkaline protease to hydrolyze the gelatin layer of spent X-ray or photographic film was investigated to recover silver [3, 7]. Certain weight (0.25 g) of used X-ray films (2.5 × 2.5 cm) were washed with distilled water, wiped with ethanol, and dried in a hot air oven at 40 °C for 30 min. Further, 20 ml of alkaline protease (140 U/ml) was used to processed X-ray films, pH was set to 9.0 using 0.1 M glycine–NaOH buffer and was incubated at 50 °C and 150 rpm. Glycine–NaOH buffer was used instead of enzyme solution as control. Turbidity of the sample (protease enzyme) increases with time due to the hydrolysis. Absorbance at 660 nm was measured until the time versus absorbance curve remains constant. The films were washed with water, dried, and the percentage of hydrolysis was measured based on the weight loss. Hydrolysis of gelatin was estimated by measuring the increase in the hydrolysate turbidity, which was associated with the release of hydroxyproline and protein.

#### Software and statistical analysis

The statistical model significance was demonstrated through *F*-test and probability value (*P*-value < 0.05**)** of the Analysis of variance (ANOVA). Additionally, the determination coefficient (*R*^*2*^) and the Adjusted *R*^*2*^ were used to achieve the fitness and reliability of the regression model [26, 27]. All experimental designs and data analysis were accomplished using Design Expert version 13.0, statistical software (Stat Ease Inc., Minneapolis, MN, USA).

## Results and discussion

### Bacterial strain and screening for proteolytic activity

**Fig. 1:**
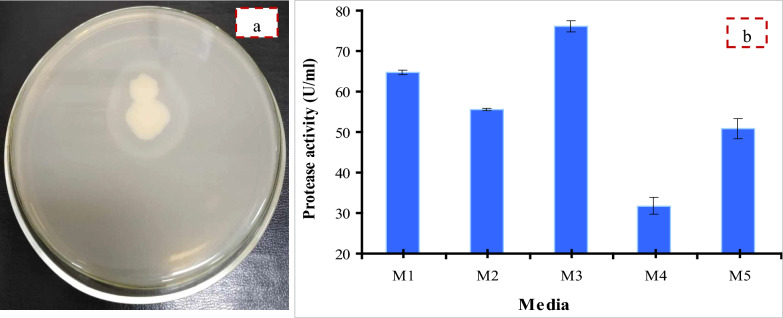
**a** Qualitative screening for proteolytic activity showing halo zone around the bacterial colony in skimmed-milk agar plate and **b** examination of diverse media for protease production by *B. licheniformis* strain-MA1

*B. licheniformis* strain-MA1 was screened for its ability to produce protease enzyme by cultivating on SMA plate medium. The results showed that *B. licheniformis* strain-MA1 can degrade casein via secretion of protease enzyme which was characterized by occurrence of a halo zone around the bacterial colonies as shown in Fig. 1a.

### Examination of diverse media for protease production

Microbial growth and enzymes production depend mainly on the nutrients in the culture medium and production conditions [13]. To determine the suitable medium for protease production, *B. licheniformis* strain-MA1 was cultured in diverse media as illustrated in Fig. 1b. M3 was the best medium for protease production (76.1 U/ml) which was 2.4-times more than M4 (31.8 U/ml). M1 and M2 exhibited enzyme production of 64.7 and 55.6 U/ml, respectively. According to the results, there were differences in protease production due to the existence of various levels of diverse metal ions that might affect the growth of bacterial strain and enzyme yield [28]. The highest production using M3 may be due to the availability of CaCl_2_ and NaCl which had a stimulating effect on the growth rates of the bacterial strain as reported by Dupree et al. [29] for lactic acid bacteria. Moreover, Boumaaza et al. [30] noticed that the most effective treatment for increasing the growth of *Botrytis cinerea* was NaCl and also CaCl_2_ stimulated their mycelial growth. Korkeala et al. [31] reported that added NaCl promoted the growth of isolated lactic acid bacteria.

### Improving protease production using multi-factorial designs

To maximize enzyme synthesis and minimize production cost, optimization of medium contents and process parameters play important roles.

### PB design

PB design was implemented to define the appropriate variables in order to promote protease production by *B. licheniformis* strain-MA1. Based on PB design, 12 experimental trials were generated and the influence of diverse combinations between the two levels (−, +) of the tested variables on protease activity (U/ml) were introduced in Table 1.

**Table 1:** Plackett–Burman (PB) design for testing variables influencing protease production by *B. licheniformis* strain-MA1

Trial	A:Wheat bran	B:Corn cob	C:Sesame cake	D:Peptone	E:Yeast extract	F:KH_2_PO_4_	G:(NH_4_)_2_SO_4_	H:FeSO_4_.7H_2_O	J:CaCl_2_	K:Culture pH	L:Incubation period	Protease activity	Predicted activity
	%	%	%	%	%	%	%	%	%		h	U/ml	U/ml
1	(+) 0.5	(−) 0	(+) 0.5	(+) 1	(+) 1	(−) 0.2	(−) 0	(−) 0.01	(+) 0.05	(−) 7	(+) 72	562.4	565.63
2	(+) 0.5	(+) 0.5	(+) 0.5	(−) 0.5	(−) 0.5	(−) 0.2	(+) 0.1	(−) 0.01	(+) 0.05	(+) 9	(−) 48	412.5	415.73
3	(+) 0.5	(+) 0.5	(−) 0	(−) 0.5	(−) 0.5	(+) 0.5	(−) 0	(+) 0.05	(+) 0.05	(−) 7	(+) 72	386.3	383.07
4	(+) 0.5	(−) 0	(+) 0.5	(+) 1	(−) 0.5	(+) 0.5	(+) 0.1	(+) 0.05	(−) 0.02	(−) 7	(−) 48	502	498.77
5	(−) 0	(−) 0	(−) 0	(−) 0.5	(−) 0.5	(−) 0.2	(−) 0	(−) 0.01	(−) 0.02	(−) 7	(−) 48	379.7	382.93
6	(−) 0	(+) 0.5	(−) 0	(+) 1	(+) 1	(−) 0.2	(+) 0.1	(+) 0.05	(+) 0.05	(−) 7	(−) 48	476.8	475.78
7	(−) 0	(−) 0	(+) 0.5	(−) 0.5	(+) 1	(+) 0.5	(−) 0	(+) 0.05	(+) 0.05	(+) 9	(−) 48	485.7	482.47
8	(−) 0	(−) 0	(−) 0	(+) 1	(−) 0.5	(+) 0.5	(+) 0.1	(−) 0.01	(+) 0.05	(+) 9	(+) 72	403.5	404.52
9	(−) 0	(+) 0.5	(+) 0.5	(−) 0.5	(+) 1	(+) 0.5	(+) 0.1	(−) 0.01	(−) 0.02	(−) 7	(+) 72	468	469.02
10	(+) 0.5	(−) 0	(−) 0	(−) 0.5	(+) 1	(−) 0.2	(+) 0.1	(+) 0.05	(−) 0.02	(+) 9	(+) 72	434.9	433.88
11	(−) 0	(+) 0.5	(+) 0.5	(+) 1	(−) 0.5	(−) 0.2	(−) 0	(+) 0.05	(−) 0.02	(+) 9	(+) 72	517.8	516.78
12	(+) 0.5	(+) 0.5	(−) 0	(+) 1	(+) 1	(+) 0.5	(−) 0	(−) 0.01	(−) 0.02	(+) 9	(−) 48	551.9	552.92

The results indicated that trial 1 exhibited the greatest protease production (562.4 U/ml) which was 7.4-fold higher than that obtained by the original medium (M3). Multiple statistical analysis was performed and the data was interpreted using the following first-order (linear) equation:

$${\text{Y}}\left( {{\text{U}}/{\text{ml}}} \right) = {465}.{13} + {9}.{\text{88A}} + {3}.{\text{76B}} + {26}.{\text{27C}} + {37}.{\text{28D}} + {31}.{\text{49E}} - {15}.{\text{51G}} - {1}0.{\text{59J}} + {2}.{\text{59K}} - {2}.{\text{98L}}$$

where Y (protease activity), A (wheat bran), B (corn cob), C (sesame cake), D (peptone), E (yeast extract), G ((NH_4_)_2_SO_4_), J (CaCl_2_), K (culture pH), and L (incubation period).

The model efficiency and the significant impact of each variable on protease production were achieved through analysis of variance (ANOVA) of PB design as displayed in Table 2. From the results, the model *F*-value (137.40) and *P*-value (0.0072) refer to the significance of the regression model. In addition, *P*-values less than 0.05 imply the model terms are significant and establish the statistical equation.

**Table 2:** ANOVA for PB design of protease production by *B. licheniformis* strain-MA1

Source	Sum of Squares	DF	Mean Square	Std. Dev	*F*-value	*P*-value	
Model	42,617.11	9	4735.23	5.87	137.40	0.0072	Significant
A-Wheat bran	1170.19	1	1170.19	0.2611	33.95	0.0282	
B-Corn cob	169.50	1	169.50	0.2611	4.92	0.1568	
C-Sesame cake	8284.51	1	8284.51	0.2611	240.38	0.0041	
D-Peptone	16,673.11	1	16,673.11	0.2611	483.78	0.0021	
E-Yeast extract	11,900.70	1	11,900.70	0.2611	345.31	0.0029	
G-(NH_4_)_2_SO_4_	2886.10	1	2886.10	0.0522	83.74	0.0117	
J-CaCl_2_	1346.20	1	1346.20	0.0157	39.06	0.0247	
K-Culture pH	80.60	1	80.60	1.04	2.34	0.2658	
L-Incubation period	106.21	1	106.21	12.53	3.08	0.2213	
Residual	68.93	2	34.46				
Cor total	42,686.04	11					

*R*^*2*^ = 0.9984, Adjusted *R*^*2*^ = 0.9911, Predicted *R*^*2*^ = 0.9419, CV = 1.26%, Adequate Precision = 34.091

DF (degree of freedom), Std. Dev. (standard deviation), Significant (*P* < 0.05), insignificant (*P* > 0.05)

Based on ANOVA data, wheat bran, sesame cake, peptone, yeast extract, (NH_4_)_2_SO_4_, and CaCl_2_ were significant variables. While the corn cob, KH_2_PO_4_, FeSO_4_·7H_2_O, culture pH, and incubation period were represented insignificant variables and had no effect on the production of protease by *B. licheniformis* strain-MA1. Also, the proportionality of the statistical model and variables effectiveness can be determined by the determination coefficient (*R*^*2*^) value when it is more than 0.9 indicating there was great correlation between predicted and recorded results [26].

As seen in Table 2, the value of *R*^*2*^ was 0.9984 which means the model can interpret 99.84% of the total variances in protease activity (U/ml). Additionally, the Adjusted *R*^*2*^-value (0.9911) and Predicted *R*^*2*^-value (0.9419) point to the suitability of the regression model. Moreover, the model reliability was demonstrated by the coefficient of variation (CV) value (CV = 1.26%) that show the good fit of statistical data. Further, Adequate Precision measures the signal-to-noise ratio where, a ratio exceeding 4 is considered desirable. In this design, the ratio of 34.091 suggests that the signal is sufficient and also implies the model is suitable for exploring the design space [32].

**Fig. 2:**
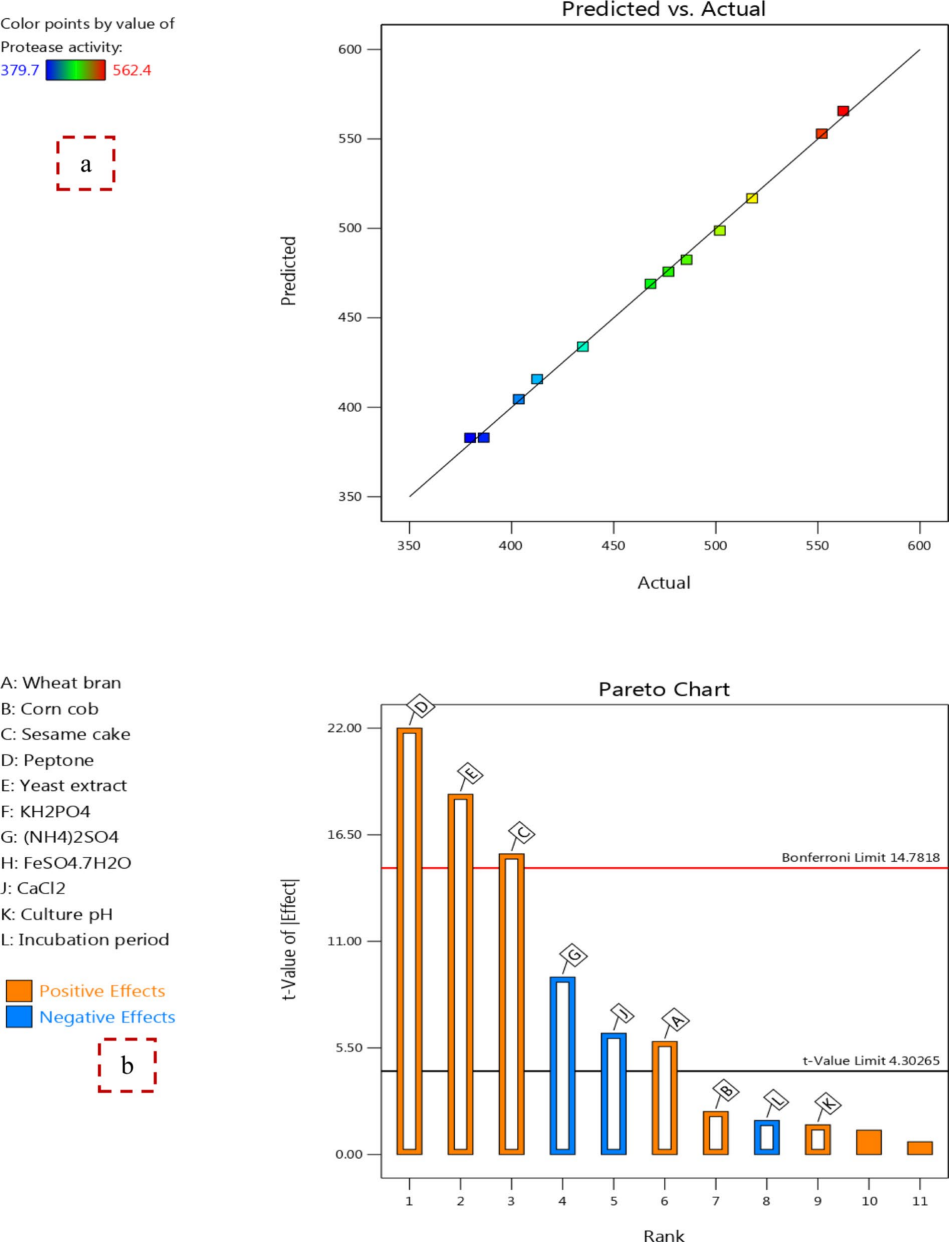
The relationship between predicted and actual values reflects their strong convergence (**a**) and the Pareto plot (**b**) of PB design for protease production by *B. licheniformis* strain-MA1

The plot of predicted and actual values of protease activity reflects the strong closing between them (Fig. 2a) which implies the great significance of regression model and high precision of the experimental trials. Besides, the prominence of PB design model was confirmed via Pareto plot that downward exhibits variables having significant effect on the production of protease by *B. licheniformis* strain-MA1. As shown in Fig. 2b, peptone, yeast extract, and sesame cake were the most significant variables followed by wheat bran that have positive effect on protease activity, while (NH_4_)_2_SO_4_, and CaCl_2_ have negative influence. Similarly, yeast extract had a positive significant effect on the production of protease by *Bacillus cereus* PW3 A using PB design model as investigated by Tennalli et al. [20]. On the other side, yeast extract, (NH_4_)_2_SO_4_, and culture pH were found to have significant impact and encourage protease production by *Lysinibacillus sphaericus* Strain AA6, whereas wheat bran was considered an insignificant variable [33].

### BB design

BB design was carried out next to the first-order screening model (PB design) to obtain the optimum levels of the selected variables (peptone, yeast extract, and sesame cake) affecting protease production by *B. licheniformis* strain-MA1 as displayed in Table 3. Numerous-regression analysis was performed and the data of BB design was affirmed using a quadratic polynomial equation as follows:

$${\text{Y}}\left( {{\text{U}}/{\text{ml}}} \right) = {367}.{7}0 + {98}.{\text{42A}} + {74}.{\text{46B}} - {25}.{\text{39C}} + {31}.{\text{23AB}} - {55}.{\text{18AC}} - {89}.{\text{75BC}} + {65}.{\text{55A}}^{2} + {9}.{\text{43B}}^{2} + {115}.{\text{98C}}^{2}$$

where Y (protease activity, predicted response), A (peptone), B (yeast extract), and C (sesame cake).

**Table 3:** Box-Behnken (BB) design for optimizing the most effective variables on protease production

Trial	A:Peptone	B:Yeast extract	C:Sesame cake	Protease activity	Predicted values
	%	%	%	U/ml	U/ml
1	(0) 1.5	(0) 1.5	(0) 0.75	365.5	367.70
2	(0) 1.5	(−) 1	(−) 0.5	344.4	354.28
3	(−) 1	(−) 1	(0) 0.75	317.8	301.01
4	(+) 2	(0) 1.5	(+) 1	574	567.09
5	(+) 2	(−) 1	(0) 0.75	441.5	435.41
6	(0) 1.5	(0) 1.5	(0) 0.75	362.3	367.70
7	(+) 2	(+) 2	(0) 0.75	630	646.79
8	(0) 1.5	(+) 2	(+) 1	462.3	452.43
9	(−) 1	(0) 1.5	(−) 0.5	414.1	421.01
10	(0) 1.5	(0) 1.5	(0) 0.75	375.3	367.70
11	(0) 1.5	(−) 1	(+) 1	470	483.00
12	(−) 1	(+) 2	(0) 0.75	381.4	387.49
13	(0) 1.5	(+) 2	(−) 0.5	695.7	682.70
14	(+) 2	(0) 1.5	(−) 0.5	732	728.21
15	(−) 1	(0) 1.5	(+) 1	476.8	480.59

The ANOVA results in Table 4 confirm the effectiveness of BB design model and all terms in the quadratic equation, so *F*-value (94.91) and *P*-value (0.0001) of the statistical model indicate that it was significant. Also, the model terms (A, B, C, AB, AC, BC, A^2^, and C^2^) exhibited *P*-values < 0.05 and were considered significant terms. Moreover, the *R*^*2*^-value (0.9942), Adjusted *R*^*2*^-value (0.9837), and Predicted *R*^*2*^-value (0.9122) demonstrate the strength of the regression model which can elucidate 99.42% of the overall variations in the enzyme activity. In addition, the CV value was relatively low (CV = 3.55%) that imply the accuracy (reliability) of the experimental design [34].

**Table 4:** ANOVA of BB design for protease production optimization

Source	Sum of squares	DF	Mean Square	Std. Dev	*F*-value	*P*-value	
Model	2.369E+ 05	9	26,325.65	16.65	94.91	< 0.0001	Significant
A-Peptone	77,499.84	1	77,499.84	0.3780	279.42	< 0.0001	
B-Yeast extract	44,357.31	1	44,357.31	0.3780	159.93	< 0.0001	
C-Sesame cake	5156.20	1	5156.20	0.1890	18.59	0.0076	
AB	3900.00	1	3900.00		14.06	0.0133	
AC	12,177.12	1	12,177.12		43.90	0.0012	
BC	32,220.25	1	32,220.25		116.17	0.0001	
A^2^	15,865.12	1	15,865.12		57.20	0.0006	
B^2^	327.99	1	327.99		1.18	0.3265	
C^2^	49,662.28	1	49,662.28		179.05	< 0.0001	
Residual	1386.80	5	277.36				
Lack of fit	1295.04	3	431.68		9.41	0.0976	Not significant
Pure error	91.76	2	45.88				
Cor total	2.383E + 05	14					

*R*^*2*^ = 0.9942, Adjusted *R*^*2*^ = 0.9837, Predicted *R*^*2*^ = 0.9122, CV = 3.55%, Adequate Precision = 31.416

DF (degree of freedom), Std. Dev. (standard deviation), Significant (*P* < 0.05), Not significant (*P* > 0.05)

Also, Adequate Precision, which is influenced by the signal-to-noise ratio, is deemed satisfactory when this ratio is greater than 4. In the BB design, the Adequate Precision value was 31.416, which signifies a strong signal and confirms that this model can be used to navigate the design space [32].

**Fig. 3:**
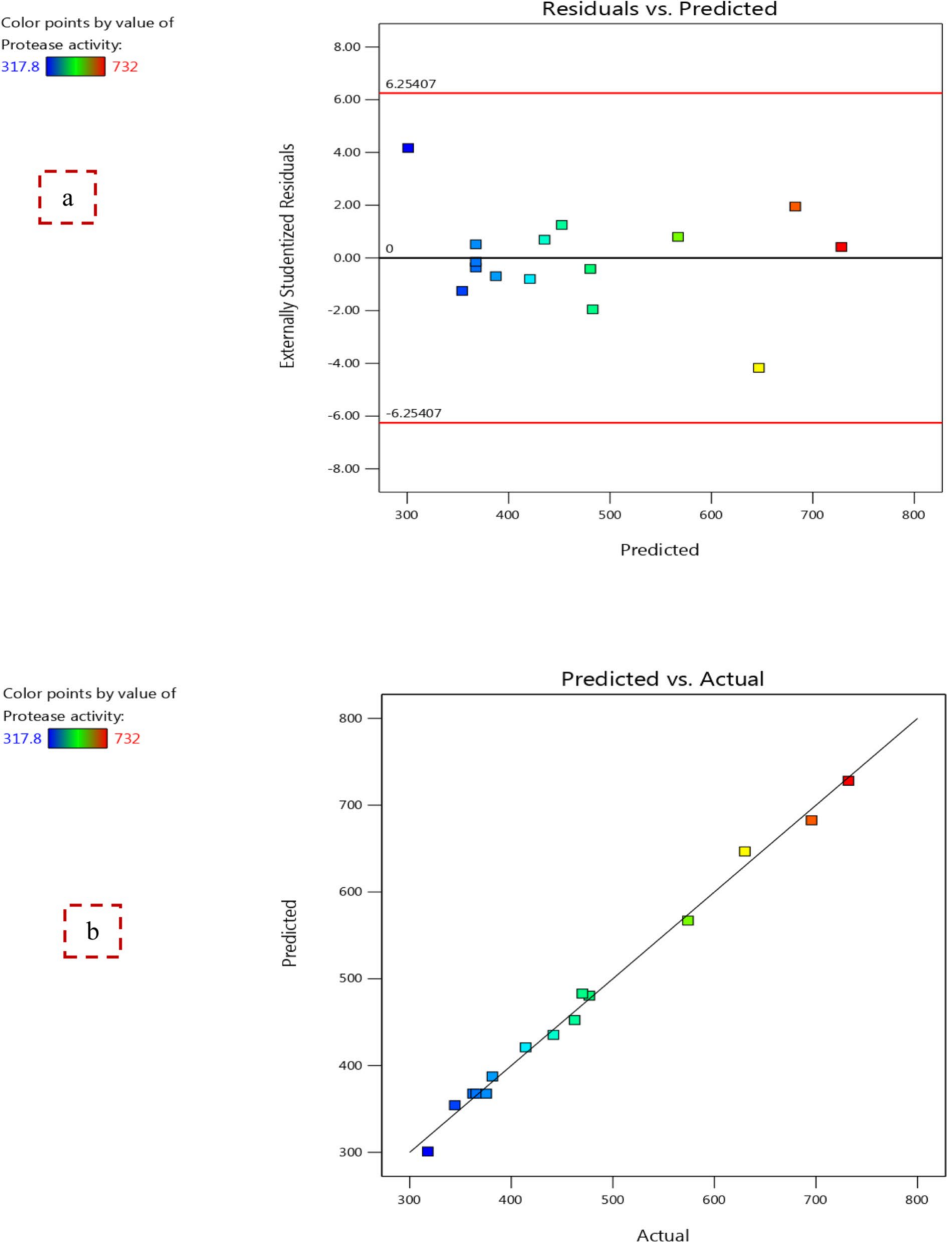
**a** Plot of residuals against predicted values and **b** The relation between predicted and actual values of BB design confirms the validation of the statistical model for protease production by *B. licheniformis* strain-MA1

The plot of residuals on the y-axis against predicted values on the x-axis shows that the residual points are scattered randomly around the horizontal zero reference indicating the great ability of BB design to optimize protease production (Fig. 3a). On the other hand, the nearness of the expected and experimental values of protease activity (Fig. 3b) exhibits the great accordance between them and confirms the validation of the statistical model.

**Fig. 4:**
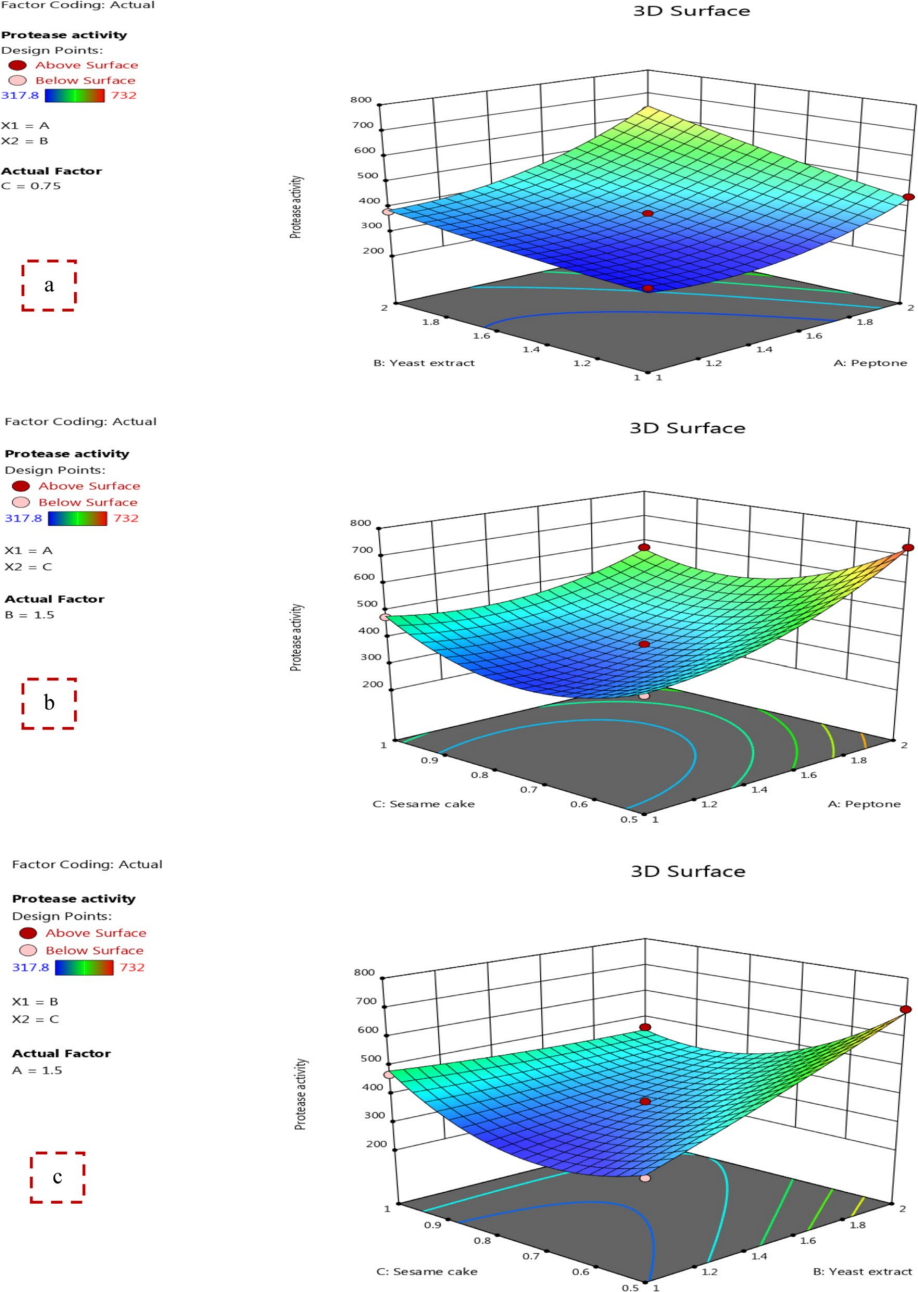
Response surface 3D displays the interaction between each two variables affecting protease activity **a** peptone and yeast extract, **b** peptone and sesame cake, **c** yeast extract and sesame cake

The interaction effects of variables on protease production were determined via the 3D surface (Fig. 4) that were established by drawing the enzyme activity on the z-axis versus two tested variables, whereas the other one was retained at its central level. Depending on the results, Fig. 4a shows the influence of peptone and yeast extract on protease activity, while sesame cake was kept at its zero level (0.75%). The highest protease activity (630 U/ml) was obtained at both high levels (2%) of peptone and yeast extract, respectively. Further, Fig. 4b illustrates the impact of peptone and sesame cake on the protease activity, while yeast extract was kept at its zero level (1.5%). The highest activity of protease (731.8 U/ml) was observed at high level (2%) of peptone and low level (0.5%) of sesame cake. Also, Fig. 4c exhibits the interaction between yeast extract and sesame cake preserving peptone at its zero level (1.5%) where, the best protease activity (695.7 U/ml) was recorded at high level (2%) of yeast extract and low level (0.5%) of sesame cake.

### Model validation and verification

To confirm the precision of the results and to verify the validation of the statistical model, a laboratory experiment was conducted based on the optimal values suggested by the BB design. The predicted optimal values were peptone 2%, yeast extract 1.5%, and sesame cake 0.5% with a predicted protease activity of 732.16 U/ml that achieves the maximum enzyme production. This value was in accordance with the experimental result that yields the highest activity of protease of 7321.8 U/ml, which demonstrates the validity of the BB design and the presence of the optimal points. At last, the maximum protease production (731.8 U/ml) by *B. licheniformis* strain-MA1 using BB design was 9.6-fold higher than that obtained from the original medium (M3). Our result was higher than that mentioned by Tennalli et al. [20] and Zhang et al. [35] who suggested that protease production from *Bacillus cereus* PW3 A and *Bacillus licheniformis* based on RSM was enhanced by 3.0- and 1.82-fold, respectively.

Also, Khan et al. [36] obtained 3.94-fold increase after statistical optimization (via PBd and BBd) of the production of protease by *Bacillus subtilis* ZMS- 2. Moreover, RSM improved the production of protease from *Lysinibacillus fusiformis* AU01 by 6-times more than the un-optimized medium [37].

The final optimized medium components (g/l) for protease production by *B. licheniformis* strain-MA1 were: wheat bran 5, sesame cake 5, peptone 20, yeast extract 15, KH_2_PO_4_ 2, FeSO_4_·7H_2_O 0.1, CaCl_2_ 0.2, NaCl 3, incubation period 72 h under 150 rpm, and culture pH 7.0.

### Biochemical characterization of protease

#### Effect of different temperatures and pHs

**Fig. 5:**
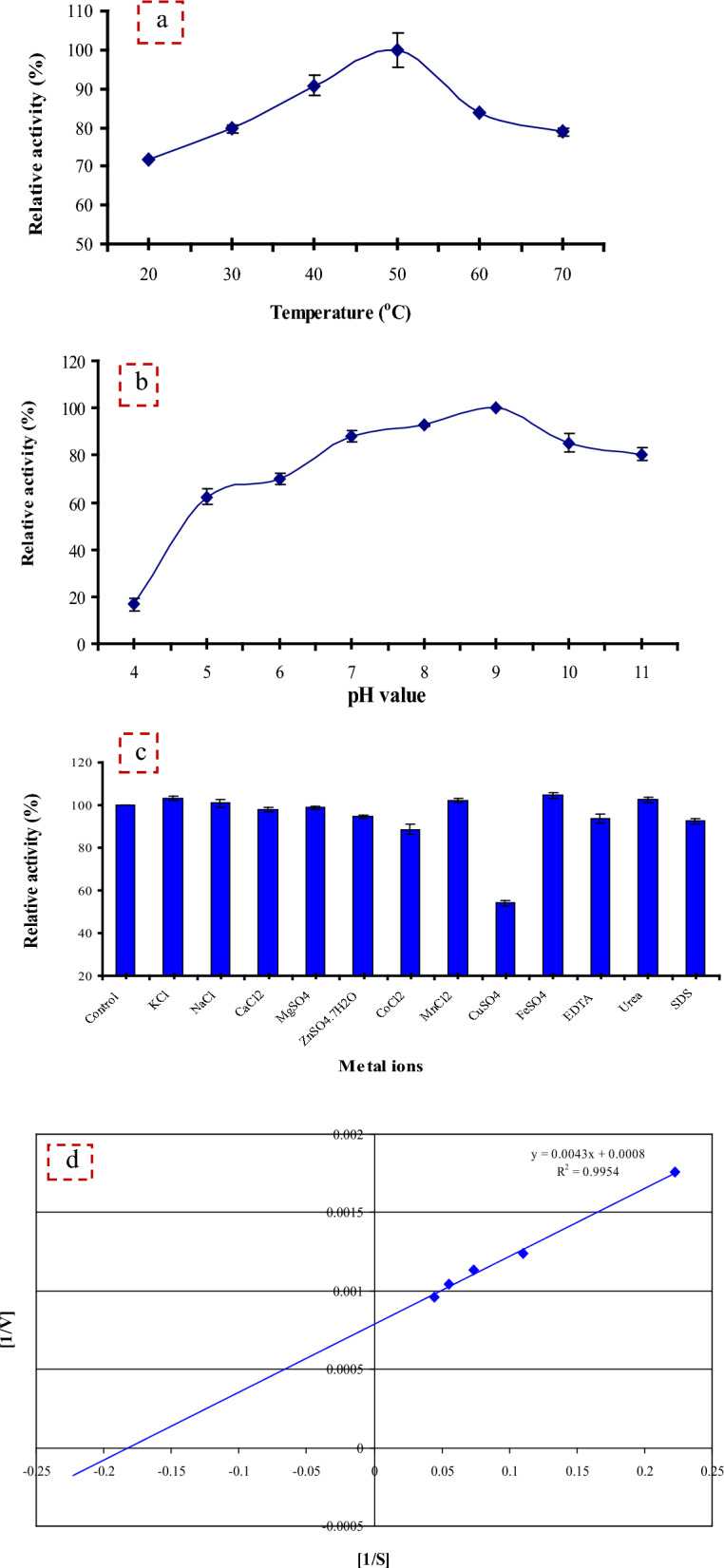
Effect of different Temperatures (**a**), pHs (**b**), and metal ions and inhibitors (**c**) on enzyme activity, and Lineweaver–Burk plot for determination of kinetic parameters of protease enzyme (**d**)

Temperature is an important factor affecting enzyme activity. A study to determine temperature optima presented in Fig. 5a showed that optimum temperature for protease activity was 50 °C. The enzyme activity decreased at 60 °C and 40 °C temperature by 16.2 and 9.2%, respectively. In general, high temperature is preferred for most enzyme activity, as high temperature increases the substrate solubility, improves rates of conversion of substrates to products, and also reduces microbial contamination [38]. Our result was similar to that reported for *B. amyloliquefaciens* crude protease [39]. Further increase in temperature results in a substantial decrease in enzyme activity due to the denaturation of the enzyme protein. Alkaline proteases used in detergent applications must be active at temperatures above 40 °C [7].

The *Ea* of the *B. licheniformis* strain-MA1 protease was calculated as 3.86 kJ/mol. This *Ea* was lower than that reported for the crude *B. amyloliquefaciens* protease (24.85 kJ/mol) [39]. The low *Ea* value of the protease indicates that it required low energy to build the activated substrate-enzyme complex, and thus it has high hydrolysis capacity.

The results illustrated in Fig. 5b showed that enzyme has lower activity when the reaction is carried out at above and below pH 9.0 and it was active at a wide pH range of 7.0–11.0. This result is consistent with that obtained by Gemechu et al. [2] who reported that alkaline proteases are enzymes active at pH in an alkaline range (7.0–11.0) with an optimum pH of 9.0.

As shown, the enzyme exhibited low activity at pH 6.0 by 29.9%. For detergent applications alkaline proteases must be active at a pH range of 9.0–12.0 [7]. The enzyme activity decreased at acidic pH confirming that the produced enzyme was an alkaline protease. The optimum pH of crude *Bacillus* sp. KU-K2 alkaline protease was 10.0 [1].

#### Effect of diverse inhibitors and metal ions

The protease used in detergent must be effective during washing and must be stable and compatible with the detergent components. Protease activity increased in presence of KCl, NaCl, MnCl, FeSO_4_, and urea (Fig. 5c). In addition, protease activity was reduced by only 6.4% in presence of EDTA as a metalloprotease. In contrast, EDTA inhibited the alkaline protease from *Bacillus* sp. KU-K2 by 46.31% [1].

### Kinetic parameters of protease enzyme

Lineweaver–Burk plot (Fig. 5d) showed that the *B. licheniformis* strain-MA1 protease had *K*_*m*_ 5.56 mg/ml and *V*_*max*_ 1250 U/ml. These results demonstrated the high catalytic efficiency of the enzyme and its high affinity for its substrate. The *V*_*max*_ of *B. licheniformis* strain-MA1 protease was greater than that obtained from *Thermomonas haemolytica* protease by tenfold [40]. Furthermore, compared with the protease from *Streptomyces* sp. LCJ12 A [41], the *B. licheniformis* strain-MA1 protease exhibited a lower *K*_*m*_ value by 13.24-fold. High *V*_*max*_ value shows the high activity of the enzyme and its potential usage in proteolytic reactions. In addition, low *K*_*m*_ value indicates the high affinity between substrate and enzyme active site and consequently high enzyme activity [7].

### Enzyme stabilities

#### pH stability

**Fig. 6:**
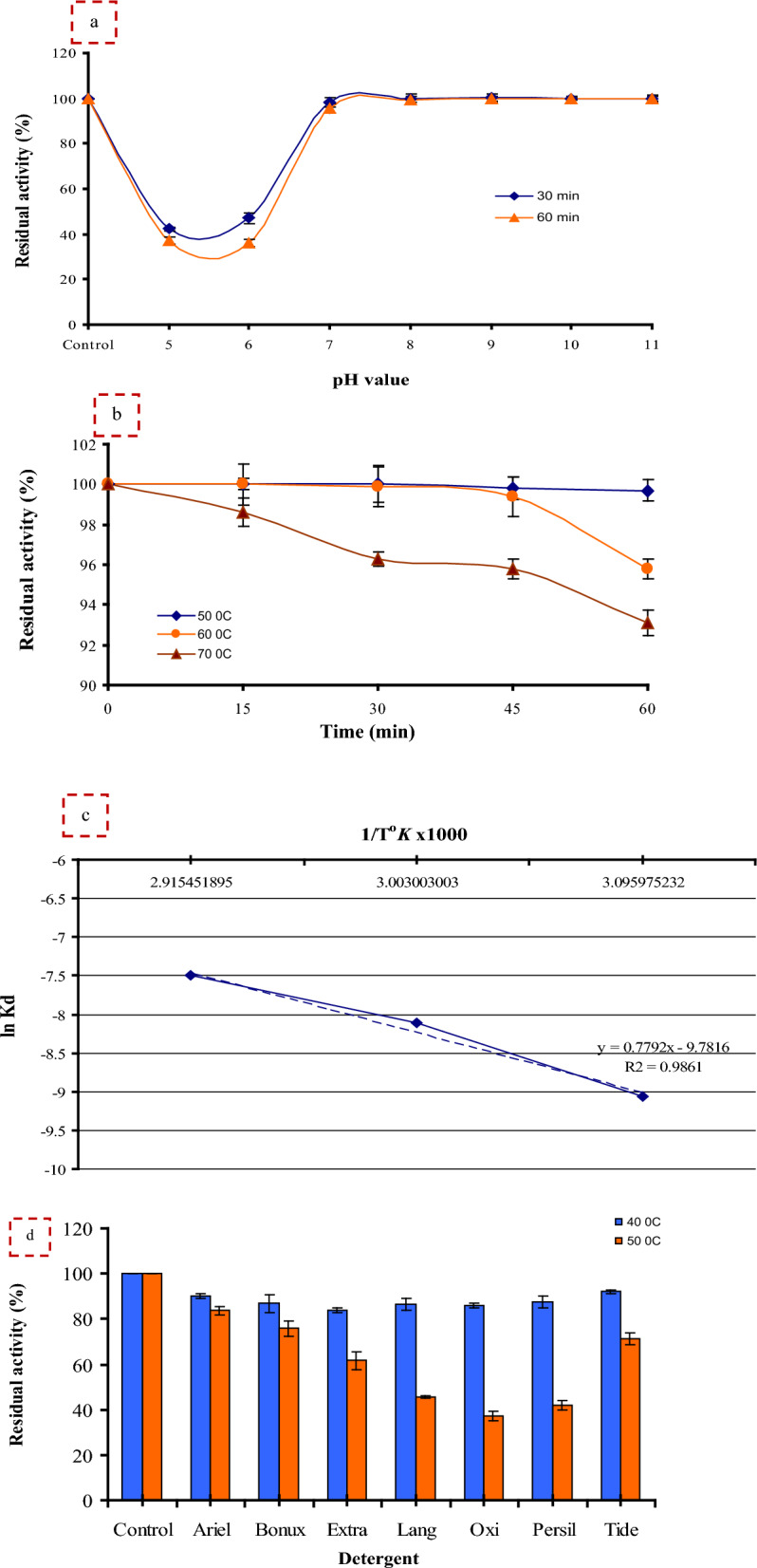
pH stability of alkaline protease (**a**), thermal stability of alkaline protease (**b**), Arrhenius plots for the thermal denaturation (*Ed*) (**c**), stability and compatibility of alkaline protease with laundry detergents (**d**)

Protease must be alkali stable to be used as a bio-additive because the pH of the detergent ranges from 9.0 to 12.0 [7]. *B. licheniformis* strain-MA1 protease activities after pre-incubation at pH 8.0 and 11.0 were stable for 30 and 60 min retained 100% of activities (Fig. 6a).

#### Thermal stability

Enzyme thermal stability is an important feature for the management of the biocatalyst to be applied industrially. The thermal inactivation of *B. licheniformis* strain-MA1 protease was studied at a temperature range of 50–70 °C. The results in Fig. 6b indicated the high thermal stability of the enzyme as it retained 99.7 and 99.4% of activity after 60 min of incubation at 50 °C and 60 °C, respectively. Furthermore, at 60 °C for 60 min the stability of *B. licheniformis* strain-MA1 protease was 1.2-fold higher than that reported by Abdel Wahab and Ahmed [7]. The crude *Pedobacter* sp. protease retained only 30% of activity after 60 min at 55 °C [42]. For industrial applications, especially laundry, the heat-stable alkaline protease mentioned here will be applicable as it retains most of its activity.

The plot of (log RA% versus time) gave straight lines indicating first-order kinetics for the protease enzyme. Thermodynamic parameters of protease thermal denaturation were summarized in Table 5. Clearly, with increasing temperature, *kd* increased and *t*_*0.5*_ and *D*-value decreased. At 60 °C and 70 °C the *kd* was higher than that at 50 °C by 2.56 and 4.75-fold, respectively.

**Table 5:** Kinetic and thermodynamic parameters of *B. licheniformis* strain-MA1protease towards thermal processes

Parameter	Temperature (°C)		
	50	60	70
*Kd* (thermal deactivation constant,/min)	1.17 X10^−4^	2.99 X10^−4^	5.55 ×10^−4^
*t*_0.5_ (half-life, h)	98.97	38.69	20.83
*D*-value (decimal reduction time, h)	328.78	128.53	69.20
*Ed (activation energy for thermal denaturation, kJ/mol)*	71.89		
*Δ*H* (enthalpy, kJ/mol)	69.21	69.13	69.05
*Δ*G* (free energy, kJ/mol)	103.64	104.33	105.78
*Δ*S* (entropy, J/mol/K)	− 0.11	− 0.11	− 0.11

The *t*_*0.5*_ is the required time at a given temperature to lose 50% from the initial enzyme activity. Therefore, in many industrial processes, it is a key economic factor as increasing *t*_*0.5*_ means increasing the enzyme thermal stability. As shown in Table 5, the calculated *t*_*0.5*_ at 50 and 60 °C were 98.97 and 38.69 h, respectively, which are 3.68 and 10.93-fold higher than that reported by Mostafa et al. [25]. At 70 °C, the *t*_*0.5*_ of the *B. licheniformis* strain-MA1 protease was 24.58-fold greater than that reported by Amin et al. [39].

The *D*-value is the time required to reduce 90% of the initial activity. The *D*-value of the protease decreased from 328.78 h to 128.53 h as the temperature increased from 50 to 60 °C. The *D*-value at 60 °C was 58.29-fold higher than that reported by da Silva et al. [43]. At 70 °C, the *D*-value was higher than that reported by Amin et al. [39] by 24.59-fold. The higher *t*_*0.5*_ and *D*-values of the produced protease indicate its higher thermal stability and thus its potential application in industrial processes.

From Fig. 6c (Arrhenius plot), the calculated *Ed* for protease thermal denaturation was 71.89 kJ/mol which was 1.88 times lower than that reported by Mostafa et al. [25]. In addition, this *Ed* was 3.97 and 1.45 times higher than that reported for other proteases [39, 43], respectively. A higher *Ed* value for an enzyme means that the enzyme requires more energy for denaturation and is therefore more resistant to heat.

Enthalpy is the total energy amount required for enzyme denaturation. Therefore, a large positive value of *∆*H* is often associated with high enzyme thermal stability. As seen in Table 5, the value of *∆*H* at 70 °C (69.05 kJ/mol) was 4.52 times higher than that reported for *B. amyloliquefaciens* protease [39]. In addition, at 50 °C *∆*H* was 19.51 kJ/mol higher than that reported by da Silva et al. [43].

A smaller or negative *∆*G* value is associated with a more spontaneous process, as the enzyme becomes less stable and undergoes denaturation more easily. In contrast, an increase in *∆*G* indicates an increase in resistance to thermal denaturation [43]. The *∆*G* of the protease at 50 °C (103.64 kJ/mol) was 1.12 times higher than that reported by da Silva et al. [43].

Entropy is the required energy per degree to go from the original to the denatured state and includes both *∆*H* and *∆*G* [25]. As shown in Table 5, at all tested temperatures, *∆*S* exhibited negative values reflecting the protease thermal stability and its industrial applicability [43]. Negative values of *∆*S* mean that at any temperature, the process under consideration was not spontaneous, but the reverse process was spontaneous, which means that denaturation is irreversible at all temperatures [43].

### Stability and compatibility with laundry detergents

The addition of enzymes to detergents has become popular in all countries that have an international market [44]. Adding proteases to laundry detergents improves cleaning by removing all protein stains [7]. The compatibility of *B. licheniformis* strain-MA1 protease with commercial laundry detergents showed that Tide at 40 °C was the most compatible detergent as the protease retained 92% of its activity (Fig. 6d). While Ariel at 50 °C was the most compatible detergent as the protease retained 83.8% of its activity. On the contrary, *Stenotrophomonas acidaminiphila* protease did not demonstrate such compatibility with Ariel as it retained 73.3% of its activity [9]. The maximum compatibility of protease enzyme was obtained with Omo which retained 97.8% and 85.5% of its activity at 25 °C and 55 °C, respectively [3]. At 40 °C, the protease showed strong compatibility with laundry detergents with activity retention in the 84–92% range. These results supported the possibility of using *B. licheniformis* strain-MA1 protease industrially as a bio-additive in detergent formulations.

### Application of protease in the gelatin hydrolysis and recovery of silver from X-ray film waste

Silver is considered one of the widely expensive and sought-after metals and is used in large quantities in various works [2]. Waste X-ray films contribute to approximately 20% of global silver manufacturing. The increasing demand for silver in the world makes it extremely difficult to obtain and manufacture new silver. Therefore, attention has recently turned to the recovery and reuse of waste X-ray films [2, 3]. The traditional physical protocol for recovering silver from waste films involves burning the films, but this leads to air pollution and an unpleasant odor. In addition, the traditional bioremediation protocol involves chemicals that can react with the film and extract silver atoms and is also expensive, harmful to the environment, and time-consuming [3]. The application of enzymes in different industries is continuously increasing, especially during the last decades [45]. Using alkaline proteases for hydrolysis of X-ray films waste to obtain pure silver recovery is an environmentally safe and economically best technology, and is acceptable to society because it does not cause an unpleasant odor [2, 3]. Alkaline proteases can perform the hydrolysis of the gelatin layer as shown in the following diagram.
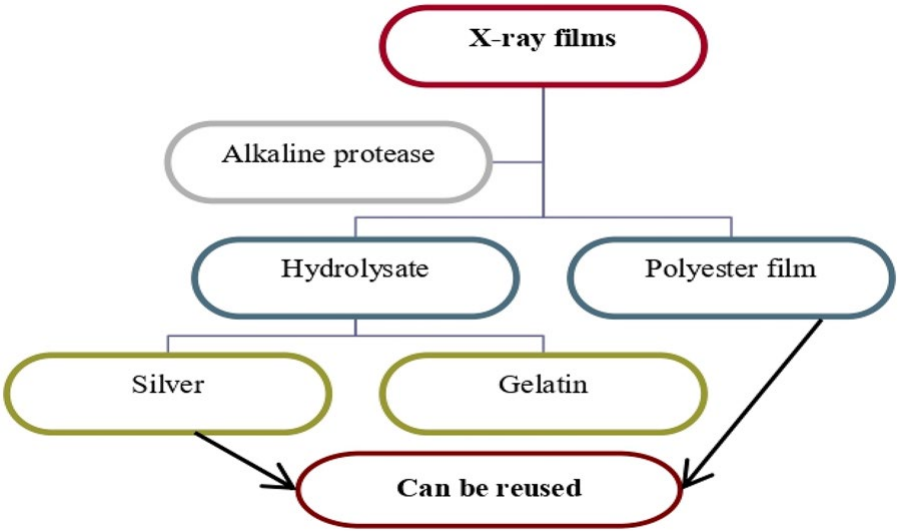


**Fig. 7:**
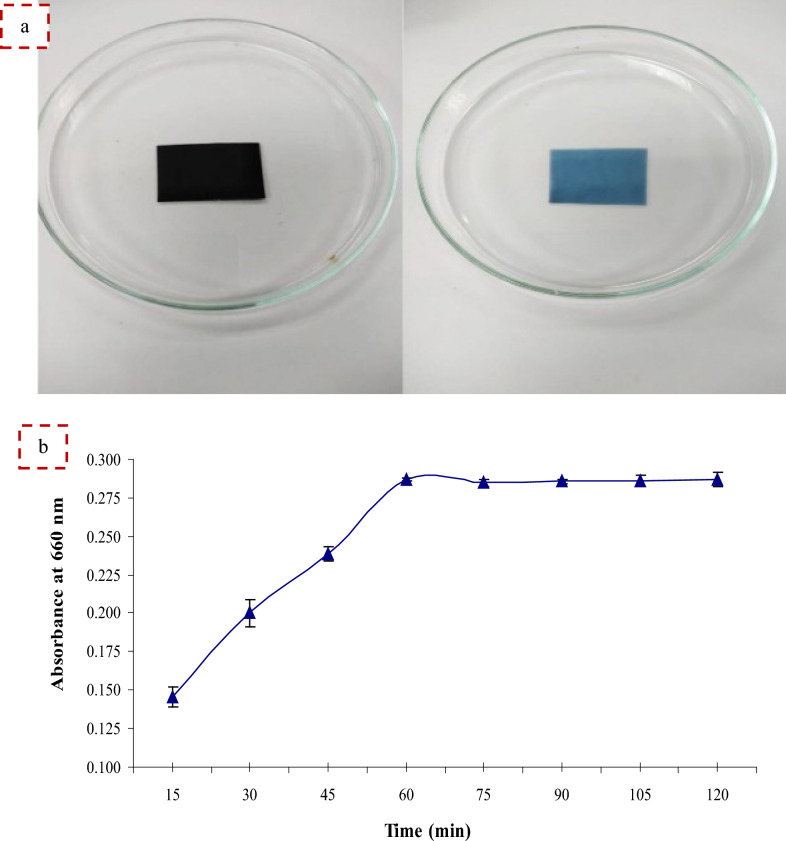
Images representing the gelatin layer of X-ray films stripped by alkaline protease enzyme (**a**) and turbidity resulting from alkaline protease hydrolysis of gelatin from used X-ray films over time (**b**)

As seen in Fig. 7a, protease successfully hydrolyzed the gelatin layer within 60 min. The present results revealed that alkaline protease can hydrolyze the gelatin layer entrapping the silver on X-ray film because it has gelatinase activity, which is an initial step for silver recovery, leading to alleviation of environmental pollution. The turbidity of the enzyme hydrolyzing gelatin from X-ray films waste increased with time and reached a maximum at 60 min (Fig. 7b).

Similarly, Abdel Wahab and Ahmed [7] obtained silver after 1 h of incubation with microbial alkaline protease. A longer exposure time of 2 h was required for gelatin hydrolysis [3]. After incubation, hydrolysis of 4.2% weight loss of the film was observed by the *B. licheniformis* strain-MA1 protease enzyme. Likewise, a 4% weight loss was observed by the enzyme extracted from *B. licheniformis* [10].

## Conclusion

According to the findings of improving protease production via multi-factorial statistical designs, peptone, yeast extract, and sesame cake emerged as the most notably significant positive factors followed by wheat bran. Statistical optimization (PB and BB) enhanced enzyme production by 9.6-fold. The low activation energy value (*Ea* 3.86 kJ/mol) of the *B. licheniformis* strain-MA1 protease suggests that it possesses a high hydrolytic ability. A low *K*_*m*_ (5.56 mg/ml) and a high *V*_*max*_ (1250 U/ml) suggest that the enzyme exhibits great catalytic efficiency and a strong affinity for its substrate. The thermal parameters (*Kd*, *t*_0.5_, *D*-value, and *Ed*) as well as the thermodynamic parameters (*Δ*H*, *Δ*G*, and *Δ*S*) of the protease enzyme showed its superior thermal stability. The higher *Δ*H* and *Δ*G* values implied that the protease enzyme exhibited great resistance to thermal denaturation. The protease remained stable after being incubated at alkaline pH (11.0) for 60 min, retaining its full activity. When EDTA was present, the protease enzyme experienced a 6.4% decrease in its activity. Protease demonstrated strong compatibility with laundry detergents, emphasizing its promise as a bio-additive. Alkaline protease can break down the gelatin layer that entrapping the silver on X-ray film and recover the silver, thus alleviating environmental pollution. Nonetheless, additional studies are necessary to utilize stable protease in other industrial applications.

## Data Availability

No datasets were generated or analysed during the current study.
